# Prevalence of intimate partner violence perpetration among military populations: A systematic review and meta-analysis

**DOI:** 10.1016/j.avb.2020.101419

**Published:** 2020

**Authors:** J. Kwan, K. Sparrow, E. Facer-Irwin, G. Thandi, N.T. Fear, D. MacManus

**Affiliations:** a Psychological Medicine Department, King's College London, Weston Education Centre, 10 Cutcombe Road, SE5 9RJ London, UK; b Department of Forensic and Neurodevelopmental Sciences, Institute of Psychiatry, Psychology and Neuroscience, King's College London, 16 De Crespigny Park, SE5 8AF London, UK

**Keywords:** Domestic violence, Spouse abuse, Intimate partner violence, Military personnel

## Abstract

Intimate partner violence (IPV) is a global health issue that impacts both civilian and military populations. Factors associated with military service may result in increased risk of IPV perpetration among Veterans and Active Duty military personnel. Six bibliographic databases were searched to identify studies that estimated the prevalence of IPV perpetration among military populations by sociodemographic and military characteristics. Where possible, random effect meta-analyses were conducted to determine pooled prevalence estimates. 42 studies were eligible for inclusion in this systematic review. 28 of these studies met the requirements for inclusion in subsequent meta-analyses. Among studies that measured past-year physical IPV perpetration, the pooled prevalence was higher among men compared to women (26% and 20% respectively). Among Veterans, there were consistently higher prevalences compared to Active Duty samples. Similarly, higher prevalences were found among studies in general military settings compared to clinical settings. Further research that considers the impact of the act(s) of IPV perpetration on the victims is needed. This, along with the use of a consistent measurement tools across studies will help to develop a stronger evidence base to inform prevention and management programs for all types of IPV perpetration among military personnel.

## Highlights

• IPV perpetration is prevalent in military populations.
• Prevalence past-year physical IPV perpetration higher among men vs women, veteran vs Active Duty, clinical vs general samples
• Need to consider impact of the act of IPV perp on victim - not just act itself.

## 1 Introduction

Research into intimate partner violence (IPV) in military populations has demonstrated that IPV is an issue of concern, and may be higher in prevalence than in civilian populations (Cronin, 1995; Griffin & Morgan, 1988; Heyman & Neidig, 1999). The World Health Organization (WHO) defines IPV as “any behaviour within an intimate relationship that causes physical, psychological or sexual harm to those in the relationship” (Krug, Dahlberg, Mercy, Zwi, & Lozano, 2002). This may include acts of physical aggression (e.g. slapping/hitting); psychological/emotional abuse (e.g. intimidation/humiliation); controlling behaviours or sexual abuse. In 2009, the National Association of Probation Officers reported that of all cases under probation in England and Wales involving a member of the UK Armed Forces, the most common conviction was for violence in a domestic setting (Napo, 2009).

Between April 2014 and March 2015, 8.2% of women and 4.0% of men reported experiencing any domestic abuse (Office for National Statistics, 2016). Of those who reported domestic abuse in the past year, the most commonly experienced type of abuse was non-sexual partner abuse (including threats, physical force, emotional or financial abuse) with 91.0% of women and 93.0% of men reporting one or more incidents. There were also significantly more reports of sexual assaults (including attempts) among women compared to men (7.0% vs 2.0% respectively) (Office for National Statistics, 2016).

The US administered the National Intimate Partner and Sexual Violence Survey (NISVS) in 2011 which asked about different forms lifetime and past year IPV victimisation. Among women, the lifetime prevalence of physical violence and psychological aggression experienced was 31.5% and 47.1% respectively. Among men, the respective prevalence rates were 27.5% and 46.5%. The prevalence of physical violence and psychological aggression in the past year was 4.0% and 14.2% respectively among women and 4.8% and 18.0% respectively among men (Breiding et al., 2014). Although past year physical violence among US women was lower compared to UK women, the other prevalence rates of physical violence and psychological aggression perpetrated by an intimate partner in the US populations was more prevalent when compared to UK populations.

As there is not an official offence category for IPV or violence against other family members in the UK, it is, difficult to examine the risk factors associated with IPV using official records. Most of the available research from the US and Canada also relies on self-reported measures of IPV. In a study of self-reported family violence (violence towards a family member) among a deployed sample of UK military personnel (Kwan et al., 2018), researchers found that the perpetration of family violence was associated with deployment in a combat role (Kwan et al., 2018). Unfortunately, this measure of violence did not distinguish between IPV and violence towards other family members.

Although there has yet to be a study that examines risk factors associated with IPV in the UK military population, research into IPV in the military in the US and in Canada have demonstrated that IPV is an issue of concern in military families. There are many possible reasons why IPV perpetration may be prevalent among military populations. Military training may encourage the use violence as a mode of conflict resolution (Adelman, 2003; Jones, 2012). It is possible that factors associated with military service could result in increased risk of IPV. These could include experiences on deployment, particularly combat exposure, which is known to be associated with violence and offending in general (MacManus et al., 2012; MacManus et al., 2013). The general demands of military life can cause military personnel to be separated from their family and support system which may impact on rates of IPV (Bradley, 2007; Rentz et al., 2006).

A large body of literature has examined the prevalence of IPV among military populations. However, methodologies used in these studies varied greatly, and provided wide ranging prevalence estimates of IPV (Brown & Joshi, 2014; Campbell et al., 2003; Cronin, 1995; Foran et al., 2011; Jones, 2012; Rosen et al., 2002). Much of the research into IPV perpetration by military personnel has been carried out using data from Veterans of conflicts prior to those in Iraq/Afghanistan (Jones, 2012; Marshall, Panuzio, & Taft, 2005; Rentz et al., 2006). These studies cannot be generalised to serving and ex-serving personnel from more recent conflicts. Today, the US and UK military are volunteer forces and more likely to have served multiple tours, compared to the US military during the Vietnam war, which was a mixture of a conscript and volunteer force and single tour deployments. Most of the research to date has been conducted in North America (i.e. the US and Canada). With other countries having their own military forces, existing research in the US may not be generalisable to these countries.

A more thorough examination of whether these prevalences vary by sociodemographic and military characteristics, as well as era of service will help to further develop support services specific to military families. Therefore, a systematic review is necessary.

This paper aims to systematically review existing literature, which has estimated the prevalence of different types of IPV perpetration in military populations. This review also aims to explore how prevalences may vary by sociodemographic and military characteristics, including gender, serving status (Active Duty or Veteran), branch of service, rank and era of service.

## 2 Methods

This review will use the WHO definition of IPV: “Any behaviour within an intimate relationship that causes physical, psychological or sexual harm to those in the relationship” (Krug et al., 2002).

### 2.1 Search strategy

Electronic searches were conducted using six bibliographic databases (EMBASE, MEDLINE, PsycINFO, Science Direct and Web of Science (including SCI, SSCI)) to identify studies that estimated the prevalence of IPV perpetration among general and clinical military populations (serving and ex-serving). Clinical populations were those which were sampled from clinical services such as Veterans Affairs (VA) Medical Clinics. Journals were hand searched, and forward citation tracking of included studies was conducted. Experts were contacted for clarification of data and methodology.

Medical Subject Headings (MeSH) and keywords were used for electronic searches (Appendix A). Terms for IPV were adapted from Cochrane protocols and previous literature reviews (Jones, 2012; Marshall et al., 2005; Ramsay et al., 2009). Terms for military personnel were taken from MeSH terms and from a list of military acronyms and abbreviations (Rubicon Planning LLC, 2016). Only English language papers were included. This review followed PRISMA reporting guidelines (Moher et al., 2009) and the protocol is registered with PROSPERO: registration CRD4201401037 (www.crd.york.ac.uk).

Studies were eligible if they: (i) included male and/or female Active Duty (AD), Reserve or ex-serving military personnel and/or their intimate partners; (ii) used a validated measure of IPV perpetration (i.e. Conflict Tactics Scale (CTS) (Straus, 2007)) or objective measures such as military records; (iii) were published in a peer-reviewed journal; (iv) used an eligible study design (e.g. randomised control trial, cohort study, cross-sectional study, etc.); and (v) reported prevalence or incidence of IPV perpetration or presented data from which these statistics could be calculated. Studies were included if the method of data collection was self-reported or partner-reported providing there was information pertaining to the perpetration of IPV by military personnel.

### 2.2 Data extraction and quality appraisal

Two reviewers screened titles and abstracts against the inclusion criteria. If it was unclear if a study met the criteria, it was carried forward to the next stage of screening. Authors were contacted if data on IPV perpetration prevalence was collected but not reported. Reviewers then assessed full texts of potentially eligible studies. Data from included papers were extracted and presented in a standardised electronic spreadsheet.

Quality appraisal was conducted by two reviewers independently using criteria adapted from validated tools (CASP UK, 2016; Downs & Black, 1998; Loney, Chambers, Bennett, Roberts, & Stratford, 2000; Saha, Chant, Wlham, & McGrath, 2005; Wing, 1994) and included items to assess study selection and measurement biases (Appendix B). Studies were categorised as high quality if they scored ≥50% on questions pertaining to selection bias. Reviewers' scores were compared and disagreements resolved before giving each study a final appraisal score.

### 2.3 Data analysis

Studies were included in the meta-analyses if they measured past-year IPV perpetration for each type of violence listed below and were disaggregated by gender. Pooled prevalence was estimated with 95% confidence intervals (CI) using random effect models. Although most of the studies used the CTS, or another validated tool adapted from the CTS, the studies did not all measure the same type of IPV.

A meta-analysis was not conducted if there were fewer than 10 studies eligible for inclusion.

### 2.4 Measures of IPV

Studies were categorised as measuring “physical IPV perpetration” if they measured acts of violence that were similar, but not limited to the items listed in the physical violence subscale of the CTS/CTS2.

Studies were categorised as measuring “severe IPV perpetration” if they measured acts of IPV with impact (clinically-significant IPV: having caused actual harm and/or injury) or specifically more severe acts listed in the physical violence subscale of the CTS/CTS2 including having beat up, threatened with or used a knife or a gun.

Studies were categorised as measuring “sexual IPV perpetration” if they measured the perpetration of sexually coercive acts against a partner, including insisting on sex, or forcing partner to have sex.

Finally, studies were categorised as measuring “psychological/emotional IPV perpetration” if they measured acts of IPV similar, but not limited to the items listed in the psychological aggression subscale of the CTS/CTS2.

Where possible, subgroup analyses were conducted for variables that were hypothesised to have an impact on the prevalence of IPV perpetration, including gender, serving status (i.e. Veteran or AD), study setting (i.e. clinical or general military setting), branch of service (i.e. Army, Air Force and Navy), and rank (enlisted, non-commissioned officer (NCO) and Officer). To examine the impact of era of service on prevalence of IPV perpetration, study samples were categorised as pre- or post-2001 (as 2001 was the beginning of the US invasion of Afghanistan), and examined study prevalences using meta-analytic subgroup analyses.

All analyses were conducted using the statistical software program, STATA, version 11.2 (StataCorp, 2009).

## 3 Results

**Fig. 1:**
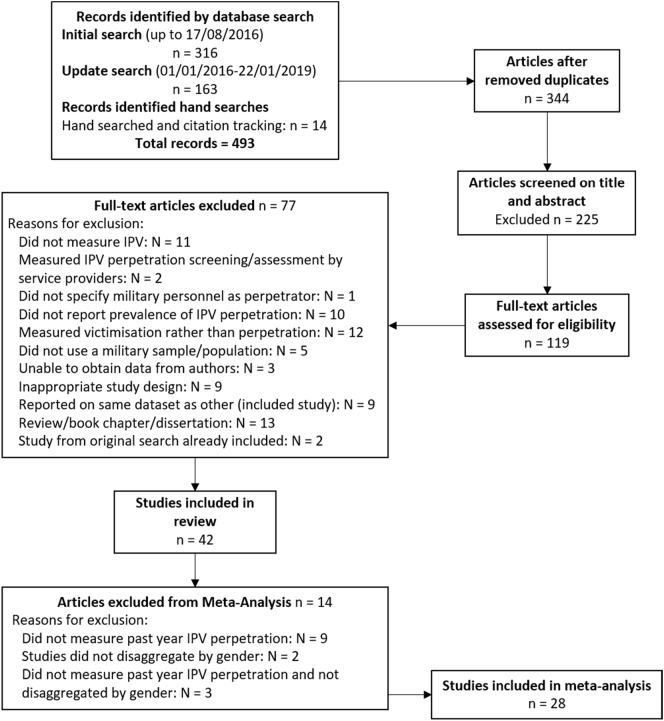
Flow diagram of screened and included papers for prevalence of IPV perpetration in military populations.

The electronic searches generated 316 references from the initial search (conducted on 17/08/2016) and 163 references from the update search (conducted on 22/01/2019). Hand searching and citation tracking identified a further 14 references. After duplicates were removed, a total of 344 references were screened for inclusion. Title and abstract screening excluded 225 references and 76 references were excluded following full-text screening, leaving 42 papers included in this review. Fourteen studies were excluded from the series of meta-analyses, leaving 28 studies (Fig. 1).

### 3.1 Key features of studies

All studies were conducted in North America (41 in the US and one in Canada) and involved a combined sample of 329,212 male and female military personnel (samples used in multiple studies were counted once). As described in Table 1, approximately a third of the studies were conducted in clinical settings and the rest were conducted in general military settings (i.e. Armed Forces communities). Most studies used the validated questionnaire CTS to measure past-year IPV, and others used similar tools adapted from the CTS.

**Table 1:** Key features of included studies.

	Number of studies
N studies (n participants)	42 (329,022)
Mean age (range)	38.6 (19.8–56.1)^a^
Gender	
Male only	20
Female only	4
Male and Female (data reported separately or together)	18
Setting	
Military clinical setting	14
General military setting	28
Era	
Pre-2001	16
Post-2001	20
Mixed	6
Status	
Active duty	19
Veteran	23
Service branch	
All regular forces	25
Army reserve	2
Army	8
Air Force	4
Navy	3
Recency of IPV	
Lifetime	2
Past 6 months	5
Past year	31
During current relationship	2
Type of domestic violence and abuse⁎	
Physical and psychological IPV	5
Any physical IPV	37
Psychological IPV	15
Sexual IPV	5
Assessment of domestic violence	
Validated instrument	37
Authors own measure	4
Military records	1
Quality appraisal score	
Low (selection bias score < 40%)	9
Moderate (selection bias score 40– 50%)	11
High (selection bias score > 50%)	22

a Mean age of 8 studies not reported.

⁎ As categories are not mutually exclusive, totals may exceed 42.

The majority (*n* = 31) of included studies reported past-year IPV perpetration (Buchholz et al., 2017; Byrne & Riggs, 1996; Dutra, de Blank, Scheiderer, & Taft, 2012; Fonesca et al., 2006; Foran et al., 2011; Forgey & Badger, 2006; Gerlock, Szarka, Cox, & Harel, 2016; Gondolf & Foster, 1991; Heavey, Homish, Goodell, & Homish, 2017; Heyman & Neidig, 1999; Hiley-Young, Blake, Abueg, Rozynko, & Gusman, 1995; Hundt & Holohan, 2012; Kar & O'Leary, 2013; Kelley, Stambaugh, Milletich, Veprinsky, & Snell, 2015; Klaw, Demers, & Da Silva, 2016; McCarroll et al., 2010; Merrill, Crouch, Thomsen, Guimond, & Milner, 2005; Newby et al., 2003; Owens et al., 2013; Pan, Neidig, & O'Leary, 1994; Rosen, Knudson, et al., 2002; Rosen, Parmley, Knudson, & Fancher, 2002c; Schmaling, Blume, & Russell, 2011; Sherman, Sautter, Jackson, Lyons, & Han, 2006; Slep, Foran, Heyman, & Snarr, 2011; Smith Slep, Foran, Heyman, & Snarr, 2010; Stander et al., 2011; Taft et al., 2009; Teten et al., 2010; Teten, Schumacher, Bailey, & Kent, 2009; Tharp, Sherman, Bowling, & Townsend, 2016). The remaining studies measuring IPV in different time periods (i.e. past 6-months and during the current relationship) were not included in the meta-analyses as they were not comparable. Study details including study design, sample size and outcomes are described in Table 2. A total of 28 of the 42 studies were included in a series of meta-analyses (Table 2).

**Table 2:** Characteristics of included studies – Prevalence of intimate partner violence perpetration among military populations.

First author, year	Sample size, setting, and gender distribution	Method of data collection	Prevalence of IPV perpetration (%)	In M-A (excluded)	Quality Appraisal Score
Buchholz et al. (2017)	810 male and female Veterans from VA hospital entering/engaged in substance use/mental health treatment	Cross-sectional study of Veterans who had completed self-report measures as part of screening for a randomised control trial Past-year Intimate Partner Violence (IPV) measured using Revised Conflict Tactics Scale (CTS2) Probable PTSD measured using PTSD Checklist for civilians (PCL—C) Probable depression measured using PHQ-9	Past-year physical IPV: *Moderate*: 187/810 = 23.1% *Severe*: 76/810 = 9.4%	No (Not dis-aggregated by gender)	Total appraisal score (T) = 24/40 Selection score (S) = 5/14 Measurement score (M) = 10/ 14
Byrne and Riggs (1996)	50 male Veterans Served in US Armed Forces during Vietnam Era and their partners (cohabiting minimum 1-year prior to participation in study	Cross-sectional study of male Vietnam Veterans Combined reported IPV: CTS PTSD symptoms: PTSD Checklist (PCL-M)	Past-year IPV: *Physical*: 17/50 = 42% *Psychological*: 50/50 = 100%	Yes	*T* = 21/40 S = 4/14 M = 8/14
Dutra et al. (2012)	89 female Vietnam Veterans	Cross-sectional study using data from the National Vietnam Veterans Readjustment Study (NVVRS) Past-year partner reports of aggression: CTS	Past-year IPV: *Physical*: 20/89 = 22.2% *Psychological* 68/89 = 76.7%	Yes	*T* = 20/40 S = 4/14 M = 8/14
Fonesca et al. (2006)	2,841 active duty (AD) US Military soldiers (Men = 2574; Women = 266) in romantic relationship, married/living with someone Processed for mobilisation Mar–Nov 2003.	Cross-sectional study of soldiers in one Army base preparing to deploy in 2003. Past-year IPV: CTS.	Past-year IPV: Overall: 449/2841 = 15.8%. Men*:* 394/2574 = 12.9% Women*:* 55/266 = 15.14%	Yes	*T* = 24/40 S = 8/14 M = 7/14
Foran et al. (2011)	42,744 AD US Air Force personnel (Men = 34,713; Women = 8031)	Cross-sectional survey collected data in 2006. Self-reported IPV and clinically significant Intimate Partner Violence (CS-IPV): CTS.	Past-year physical IPV: *IPV*: Men*:* 4478/34712 = 12.9% Women*:* 1216/8031 *=* 15.14% *CS-IPV*: Men*:* 1618/34713 = 4.66% Women*:* 268/8031 = 3.34%.	Yes	*T* = 31/40 S = 10/14 M = 12/14
Forgey and Badger (2006)	248 enlisted female personnel married to civilian spouse and in military for at least 6-months	Cross-sectional study of large Army installation during the summer of 2001 Self-reported violence: CTS	Past-year minor IPV: *Physical*: 75/248 = 30.2% *Sexual*: 49/248 = 19.8% *Psychological*: 222/248 = 89.5% Past-year severe IPV: *Physical*: 28/248 = 11.3% *Sexual*: 1/248 = 0.4% *Psychological*: 80/248 = 32.3%	Yes	*T* = 25/40 S = 6/14 M = 9/14
Gerlock et al. (2016)	441 male Veterans in treatment for PTSD in VA medical centres in an intimate heterosexual relationship (minimum 1-year)	Interview and self-report questionnaire by personnel with PTSD Past-year IPV perpetration: comparing Veteran and spouse/partner reports and examination of responses on the Abusive Behaviour Inventory questionnaire PTSD severity: Clinician administered PTSD scale (CAPS)	Past-year physical IPV: 117/441 = 26.5%	Yes	*T* = 27/40 S = 9/14 M = 8/14
Gondolf and Foster (1991)	218 male patients admitted to inpatient alcohol rehabilitation program for military Veterans over 6-month period (1988–1989)	Self-administered questionnaire Alcohol dependency: Short Michigan Alcoholism Screening Test Perpetration of wife assault in the Past-year: CTS	Past-year IPV: *Physical*: 85/218 = 39% *Severe assault*: 44/218 = 20%	Yes	*T* = 23/40 S = 6/14 M = 7/14
Heavey et al. (2017)	257 US Army Reserve/National Guard soldiers with combat exposure and their partners	Cross-sectional survey of national guard and reserve soldiers Past-year IPV: CTS Combat exposure: completion of the Deployment Risk and resilience Inventory-2 PTSD: PTSD Checklist	Past-year physical IPV *Moderate:* Men: 37/246 = 17.5% Women: 8/33 = 24.2% *Severe:* Men: 14/246 = 5.7% Women: 3/33 = 9.1% Past-year sexual IPV*:* Men: 37/246 = 14.9% Women: 4/33 = 12.1%	Yes	T = 23/40 S = 7/14 M = 9/14
Heyman and Neidig (1999)	27,502 married, male, AD Army personnel	Cross-sectional study of large, reasonably representative of US Army installation between 1990 and 1994 Self-reported IPV: CTS	Past-year physical IPV: *Moderate*: 2970/27502 = 10.8% *Severe*: 688/27502 = 2.5%	Yes	*T* = 32/40 S = 13/14 M = 9/14
Hiley-Young et al. (1995)	177 male Vietnam combat VeteranVeteran inpatients	Cross-sectional study of patients admitted to VA hospital PTSD units between 1986 and 1987 Combat experience and witnessing/participating in abusing violence measured by the Vietnam Era Stress Inventory part 2 and 3 respectively PTSD: subscale of MMPI Violence with wife: unpublished measure	Past-year physical IPV: 102/177 = 57.6%	Yes	*T* = 18/40 S = 5/14 M = 6/14
Hundt and Holohan (2012)	264 male and female mixed-era Veterans attending outpatient mental health Veteran Affairs hospital clinic	Case-file review of mixed-era Veterans presenting for outpatient mental health treatment at Veteran's hospital clinic Past-year physical IPV: hospital routine intake question “Have you punched, grabbed, slapped, or punched your partner in the past-year?” Scored positive for physical violence if answered “yes”. PTSD: PTSD Checklist Civilian (PCL—C) Depression: Beck Depression Inventory	Past-year physical IPV: 43/254 = 16.3% Men*:* 39/232 = 16.8% Women*:* 4/22 = 18.2%	Yes	T = 25/40 S = 5/14 M = 9/14
Kar and O'Leary (2013)	110 male OEF/OIF Veterans, deployed at least once and in relationship with same partner prior to deployment	Cross-sectional survey of Veteran recruited from Northport Veterans Affairs Medical Center medical records database (Jan–May 2010) PTSD: PTSD Checklist Military (PCL-M) Physical IPV perpetration: CTS2	Past-year physical IPV: 34/110 = 30.9%	Yes	*T* = 26/40 S = 6/14 M = 10/14
Kelley et al. (2015)	72 Navy members (Men = 58; Women = 18) in relationships	Cross-sectional survey of Navy members anticipating deployment within 2 months. IPV: CTS2, short version	Past-year physical IPV: 17/72 = 23.6%	No, not disaggregated by gender	T = 26/40 S = 8/14 M = 9/14
Klaw et al. (2016)	131 male post-9/11 Veteran in college and in relationships	Cross-sectional online survey of post-9/11 Veterans recruited from California State Universities and Community Colleges Alcohol use: AUDIT; Drug use: Drug Abuse Screening Test (DAST) Relationship satisfaction: Relationship Assessment Scale (RAS). Lifetime IPV: CTS	Lifetime IPV: *Physical:* Threatened to hit/throw something at partner: 16/131 = 12% Grabbed partner: 16/131 = 12% *Sexual:* Coerced partner to have sex with them: 37/131 = 28% *Psychological:* Insulted/swore at partner: 89/131 = 68% Shouted at partner: 88/131 = 67% Destroyed something of partner's: 13/131 = 10%	Yes	*T* = 22/40 S = 6/14 M = 7/14
LaMotte, Taft, Weatherill, Scott, and Eckhardt (2014)	65 male OIF/OEF combat Veterans and their partners	Cross-sectional study, Veterans exposed to combat and married/living with partner for a minimum of 6-months. Intimate partner aggression (IPA) over past 6-months: CTS2	Past 6-months IPV: *Physical*: 13/65 = 20% *Psychological*: 58/65 = 89.2%	No (Not past-year IPV)	T = 27/40 S = 9/14 M = 6/14
LaMotte, Taft, Reardon, and Miller (2014)	239 mixed-era couples cohabiting for 12 months and exposed to minimum one traumatic event, not in previous study (Teten et al., 2009)	Cross-sectional survey at VA Boston Healthcare System and New Mexico VA Healthcare system IPA over past 6-months: CTS2	Past 6-months IPV: *Physical*: 57/239 = 23.8% *Psychological*: 225/239 = 93.7%	No (Not past-year IPV)	T = 27/40 S = 9/14 M = 6/14
LaMotte, Taft, Weatherill, and Eckhardt (2017)	92 male Veterans cohabiting with partner (minimum past 6-months) Exposed to combat and reported exposure to minimum one event meeting PTSD Criterion A	Cross-sectional study of returning Veteran recruited from the greater Boston area IPA over past 6-months: CTS2 PTSD: CAPS	Past 6-months IPV: *Physical*: 27.5% *Psychological*: 93.4%	No (Not past-year IPV)	T = 23/40 S = 4/14 M = 9/14
McCarroll et al. (2003)	1,025 AD, male, Army soldiers	Cross-sectional survey of deployed and non-deployed Army soldiers (deployed for 6-months to Bosnia) Moderate and Severe physical violence measured pre- and post-deployment for deployed and non-deployed participants using CTS - Pre-deployment: pre-Sep 1998 - Post-deployment: Apr 1999–Jun1999 (time of survey)	Pre-deployment physical IPV: 109/1025 = 10.6% Post-deployment physical IPV: 74/1025 = 7.0%	No (Not past-year IPV)	T = 32/40 S = 12/14 M = 11/14
McCarroll et al. (2010)	26,835 married, AD Army men and women at 47 Army installations (25,520 men and 1315 women)	Cross-sectional survey of representative sample of AD Army between 1990 and 1994 Past-year IPV: CTS	Past-year physical aggression: *Moderate:* Men: 4670/25520 = 18.3% Women: 318/1315 = 24.2% *Severe:* Men: 1327/25520 = 5.2% Women: 105/1315 = 8.0%	Yes	T = 32/40 S = 12/14 M = 10/14
Merrill et al. (2005)	963 male and female US Navy Recruits at RCT (Men = 421; Women = 542)	Cross-sectional survey at a Recruit Training Command, Great Lakes, Illinois Past-year severe IPV: CTS	Past-year severe physical IPV: Overall: 134/963 = 14% Men: 68/421 = 16% Women: 66/542 = 12%	Yes	T = 26/40 S = 7/14 M = 10/14
Newby et al. (2003)	1,185 female US Army soldiers married to employed vs unemployed civilian husbands	Cross-sectional survey administered to married, AD Army personnel Moderate and severe violence: CTS	Past-year physical aggression *Moderate*: 287/1185 = 24.2% *Severe*: 95/1185 = 8.0%	Yes	T = 26/40 S = 7/14 M = 10/14
Owens et al. (2013)	125 male and 8 female Veterans attending VA medical centre	Cross-sectional survey of male and female Veterans seeking treatment in either the Posttraumatic Stress Program or Substance Use Disorders Program Past-year physical violence and psychological abuse against a current partner: CTS PTSD symptoms: PCL-M	Past-year IPV: *Physical aggression and psychological abuse:* Men: 21/125 = 16.8% Women: 1/8 = 12.5% *Psychological abuse*: Men: 83/125 = 66.4% Women: 5/8 = 62.5%	Yes	T = 24/40 S = 4/14 M = 9/14
Pan et al. (1994)	15,023 male AD Army soldiers from 38 US Army bases	Cross-sectional survey of randomly selected men on 38 Army bases in the US Tactics to resolving marital conflicts (not, mildly or severely physically aggressive): Modified CTS (adapted from CTS)	Past-year physical aggression: *Overall*: 3549/11870 = 29.9% *Mild*: 2896/11870 = 24.4% *Severe*: 665/11870 = 5.6%	Yes	T=/2740 S = 10/14 M = 10/14
Petrick, Rosenberg, and Watson (1983)	101 New admissions and readmissions to a Veterans Administration Medical Centre	St Cloud Veterans Administration psychiatric and chemically dependent inpatients Classified as “Batterer” if ever physically hurt woman (married to/living with) or engaged in any of 14 specific forms of violence	Lifetime physical IPV: 54/101 = 53.5%	No (Not past-year IPV)	*T* = 16/40 S = 3/14 M = 6/14
Portnoy et al. (2018)	187 women from a national study of women Veterans' IPV-related health needs	Cross-sectional study of data from a 2016 web-based survey IPV in past 6-months: CTS2 and modified *E*-HITS screening tool (developed by study team)	Past 6-month IPV: 31/187 = 16.6% *Physical*: 51.6% *Sexual*: 29% *Psychological*: 90.3%	No (Not past-year IPV)	T = 22/40 S = 7/14 M = 9/14
Rabenhorst et al. (2013)	156,296 married AD Air Force personnel deployed to OEF/OIF (Men =133,079; Women =23,217)	Data linkage study 2001–2008 examining instances of substantiated spouse abuse on Family Advocacy System of Records (FASOR) before and after deployment. Personnel have experienced combat-related deployment IPV during study period: substantiated incident of spouse physical/emotional abuse in FASOR database	Overall IPV during study period (2001–2008): 3524/156296 = 2.25% Men*:* 3165/133079 = 2.03% Women*:* 359/23217 = 0.23%	No (Not past-year IPV)	T = 24/40 S = 8/14 M = 8/14
Rosen, Knudson, et al. (2002)	648 married male AD Army personnel stationed at an installation in Alaska	Self-report questionnaires administered in the Summer 1998 Past-year IPV measured using Military Conflict Tactics Scale (MCTS)	Past-year physical IPV: 205/648 = 31.6% *Minor*: 148/648 = 22.5% *Moderate/severe*: 59/648 = 9.1%	Yes	T = 23/40 S = 6/14 M = 7/14
Rosen, Parmley, Knudson, and Fancher (2002b)	576 AD Army personnel stationed at an installation in Alaska (Men = 477; Women = 99)	Self-report questionnaires administered in the Summer 1998 Past-year IPV: MCTS	Past-year physical IPV: *Minor:* Men*:* 143/446 = 32% Women*:* 35/91 = 38% *Moderate/Severe:* Men*:* 54/446 = 12% Women*:* 16/91 = 17%	Men Yes)	T = 23/40 S = 6/14 M = 8/14
Sayers, Farrow, Ross, and Oslin (2009)	134 male and female military Veterans who served in Iraq or Afghanistan after 2001	Cross-sectional study of clinical population at the Philadelphia VA Medical Clinic Any IPV in past 6-months: series of questions drawn from CTS	Past 6-months overall IPV: 81/134 = 60%	No (Not past-year IPV)	T = 26/40 S = 5/14 M = 12/14
Schmaling et al. (2011)	546 male, married AD US Army reserves deployed to Operation Iraqi Freedom (OIF).	Longitudinal questionnaire based study of reserve personnel pre and post-mobilisation at one Army base in 2003. Past-year IPV: CTS.	Past-year overall IPV: 65/546 = 13.5%.	Yes	T = 22/40 S = 6/14 M = 9/14
Sherman et al. (2006)	179 male Veterans in committed relationship with a cohabiting female partner	Cross-sectional study of couples seeking relationship therapy at the Family Mental Health Program Mental health issue– combat related PTSD, major depressive disorder, adjustment disorder: medical records Past-year physical violence: CTS	Past-year physical IPV: *Overall*: 77/179 = 43% *Severe*: 26/179 = 14.5%	Yes	T = 25/40 S = 7/14 M = 9/14
Smith Slep et al. (2010)	42,744 AD US Air Force personnel (Men = 34,713; Women = 8031) in relationships	Cross-sectional survey of AD Air Force members completing the 2006 Community Assessment (anonymous survey conducted at 82 sites worldwide) IPV: measure similar to CTS Alcohol: AUDIT-10	Past-year physical IPV: Men*:* 1736/34713 = 5% Women*:* 723/8031 = 9%	Yes	*T* = 28/40 S = 10/14 M = 10/14
Smith Slep, Foran, Heyman, and Snarr (2011)	42,744 AD US Air Force personnel (Men = 34,713; Women = 8031) in relationships	Cross-sectional survey of AD Air Force members completing the 2006 Community Assessment (anonymous survey conducted at 82 sites worldwide) IPV: measure similar to CTS Alcohol: AUDIT-10	Past-year physical CS-IPV: Men*:* 310/28,758 = 1.1% Women*:* 93/6,633 = 1.4%	Yes	T = 28/40 S = 10/14 M = 10/14
Stander et al. (2011)	147 male and 230 female AD Navy personnel who were married/cohabiting	Longitudinal study using data from large survey of Navy Recruits Past-year intimate partner aggression (IPA): CTS	Past-year physical aggression: *Overall*: Men: 22/147 = 15% Women: 7/230 = 32% *Mild*: Men: 15/147 = 10% Women: 41/230 = 18% *Severe*: Men: 7/147 = 5% Women: 32/230 = 14% *Verbal*: Men: 88/147 = 60% Women: 117/230 = 51%	Yes	T = 27/40 S = 7/14 M = 9/14
Taft, Street, Marshall, Dowdall, and Riggs (2007)	60 male Vietnam combat Veterans who had served in Vietnam theatre between 1964 and 1973 In romantic relationship (minimum 1-year)	Recruited from 1997 to 1998 in a Department of VA Medical Centre PTSD: CAPS IPV during current relationship: CTS- 9-item Physical Assault and 7-item Psychological Aggression subscales	IPV during the current relationship: *Physical assault*: 24/60 = 40% *Psychological aggression*: 55/60 = 91%	No (Not past-year IPV)	T = 23/40 S = 6/14 M = 8/14
Taft et al. (2009)	161 male Veterans accessing VA medical clinic	Cross-sectional survey of male Veterans attending a Veteran healthcare service for PTSD between 2003 and 2008 Past-year physical violence and psychological abuse against partner: CTS-R PTSD symptoms: CAPS	Past-year IPV: *Physical violence*: 53/161 = 32.9% *Psychological abuse*: 147/161 = 91.3%	Yes	*T* = 29/40 S = 8/14 M = 9/14
Teten et al. (2009)	92 male Vietnam and/or Iraq/Afghanistan Veterans accessing a VA medical centre outpatient trauma recovery clinic	Past-year sexual aggression and past 6-month verbal/physical aggression: CTS2 and Psychological maltreatment of women inventory (PMWI)	Past-year IPV: *Physical assault*: 36/92 = 39.1% *Sexual aggression*: 37/92 = 40.2% *Psychological aggression*: 44/92 = 80.0%	Yes	T = 25/40 S = 8/14 M = 10/14
Teten et al. (2010)	86 male Vietnam and OEF/OIF Veterans from a VA medical center outpatient PTSD clinic	Cross-sectional study of Veterans who had completed routine screening for PTSD Past-year IPV: CTS2	Past-year IPV *Physical* OEF/OIF: 27/57 = 47.4% Vietnam: 9/28 = 32.1% *Psychological* OEF/OIF: 50/57 = 87.7% Vietnam: 23/28 = 82.1%	Yes	T = 29/40 S = 8/14 M = 10/14
Tharp et al. (2016)	99 US male Veterans deployed to Iraq and/or Afghanistan and their female partners	Cross-sectional study of male Veterans in committed relationship with cohabitating female partner seeking therapy at the Family Mental Health Program between August 2004–June 2012 PTSD diagnoses made based on diagnostic interviews conducted by VA psychologist/psychiatrist Demographic and clinical variables measured using 22-item measure created by authors. IPV: combination of items from CTS and CTS-2	Past-year IPV: *Physical assault*: 34/99 = 34.4% *Sexual aggression*: 27/99 = 27.6% *Verbal aggression*: 99/99 = 100%	Yes	T = 25/40 S = 6/14 M = 8/14
Waliski, Blevins, Spencer, Roca, and Kirchner (2013)	OEF/OIF Veterans of National Guard units in Arkansas (Men = 288; Women = 24)	Cross-sectional survey of Veterans participating in a quasi-experimental evaluation of acceptance and commitment therapy Depression: PAQ-9 PTSD: PCL-C Substance use/abuse: AUDIT Lifetime physical violence against partner: CTS	Lifetime physical IPV: Men: 50/288 = 17.4% Women: 8/24 = 33.3%	No (Not past-year IPV)	T = 26/40 S = 6/14 M = 11/14
Zamorski and Wiens-Kinkaid (2013)	1745 AD Canadian Regular Forces Personnel (Men = 1,017, Women = 728).	Cross-sectional study using stratified randomised sample of regular Canadian Armed Forces personnel, data collected 2008–2009. Self-reported IPV perpetration over course of current relationship: questions from Canadian General Social Survey similar to CTS.	IPV during the current relationship: *Physical/sexual violence*: Men: 70/984 = 9.5% Women: 55/709 = 9.0% *Emotional/financial:* Men: 161/984 = 19.3% Women: 118/709 = 18.7%	No (Not past-year IPV)	*T* = 32/40 S = 12/14 M = 12/14

### 3.2 Physical IPV perpetration

**Fig. 2:**
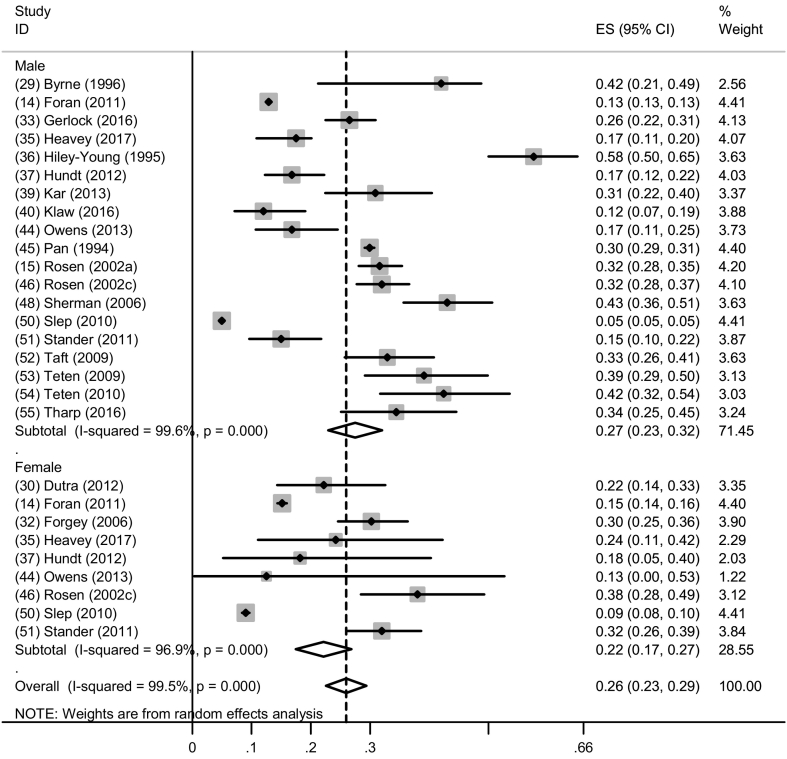
Forest plot of past year physical IPV perpetration by gender.

Thirty studies measured physical IPV perpetration among military populations with estimates ranging from 5.0%–57.6% (Buchholz et al., 2017; Byrne & Riggs, 1996; Dutra et al., 2012; Foran et al., 2011; Forgey & Badger, 2006; Gerlock et al., 2016; Heavey et al., 2017; Hiley-Young et al., 1995; Hundt & Holohan, 2012; Kar & O'Leary, 2013; Kelley et al., 2015; Klaw et al., 2016; LaMotte et al., 2017; LaMotte, Taft, Reardon, & Miller, 2014; LaMotte, Taft, Weatherill, et al., 2014; Owens et al., 2013; Pan et al., 1994; Petrick et al., 1983; Portnoy et al., 2018; Rosen, Knudson, et al., 2002; Rosen, Parmley, et al., 2002c; Sherman et al., 2006; Smith Slep et al., 2010; Stander et al., 2011; Taft et al., 2007; Taft et al., 2009; Teten et al., 2009; Teten et al., 2010; Tharp et al., 2016; Waliski et al., 2013; Zamorski & Wiens-Kinkaid, 2013). Fifteen studies were suitable for inclusion in a meta-analysis for past-year physical IPV with subgroup analysis by gender (Foran et al., 2011; Forgey & Badger, 2006; Gerlock et al., 2016; Heavey et al., 2017; Hiley-Young et al., 1995; Hundt & Holohan, 2012; Kar & O'Leary, 2013; Klaw et al., 2016; Owens et al., 2013; Pan et al., 1994; Rosen, Knudson, et al., 2002; Rosen, Parmley, et al., 2002c; Sherman et al., 2006; Smith Slep et al., 2010; Stander et al., 2011; Taft et al., 2009; Teten et al., 2009; Teten et al., 2010; Tharp et al., 2016) (Fig. 2). Overall pooled past-year physical IPV prevalence was 26.0% (95% CI: 23.0%–29.0%).

When separated by gender, nineteen studies provided estimates of past-year physical IPV for men and nine provided estimates for women (Fig. 2). The prevalence of past-year physical IPV among men ranged from 5.0%–58.0% and the pooled prevalence was 27.0% (95% CI: 23.0%–32.0%, I^2^ = 99.6%, *p* < 0.001). Two studies among samples of male Veterans in a clinical setting reported much higher prevalences (43.0% and 58.0%) compared to the other included studies (in non-clinical and AD samples) (Gerlock et al., 2016; Pan et al., 1994). Among women, the prevalence of past-year physical IPV ranged from 9%–38% and the pooled prevalence was 22.0% (95% CI: 17.0%–27.0%, I^2^ = 96.9%, p < 0.001). One study of a large Air Force population in general military settings reported much lower prevalences among both male and female AD Air Force personnel (5% and 9% respectively) (Smith Slep et al., 2010).

Although pooled prevalence estimates for past-year physical IPV perpetration were higher among men compared to women (27.0% vs. 22.0%), six studies comparing past-year self-reported physical IPV among women and men within the same study reported higher prevalence among women compared to men (15.1% vs. 12.9% (Foran et al., 2011); 18.2% vs. 16.8% (Hundt & Holohan, 2012); 24.2% vs. 18.3% (McCarroll et al., 2010); 9.0% vs. 5.0% (Smith Slep et al., 2010); 32.0% vs. 15.0% (Stander et al., 2011); 24.2% vs. 17.5% (Heavey et al., 2017)).

Prevalence estimates varied among studies that measured IPV perpetration across different time frames: 17.9% of a general military sample of male US Army soldiers reported perpetrating physical IPV in the past 3-months (McCarroll et al., 2003) and 17.4% male and 33.3% female OEF/OIF National Guard Veterans reported lifetime physical IPV perpetration (Waliski et al., 2013). Several studies measured physical IPV perpetration in the past 6-months (LaMotte et al., 2017; LaMotte, Taft, Reardon, & Miller, 2014; LaMotte, Taft, Weatherill, et al., 2014; Portnoy et al., 2018), where two reported prevalences of 20.0% and 27.5% among samples of male Veterans (LaMotte et al., 2017; LaMotte, Taft, Weatherill, et al., 2014), and another reported a prevalence of 51.6% among women Veterans (Portnoy et al., 2018).

### 3.3 Severe IPV perpetration

**Fig. 3:**
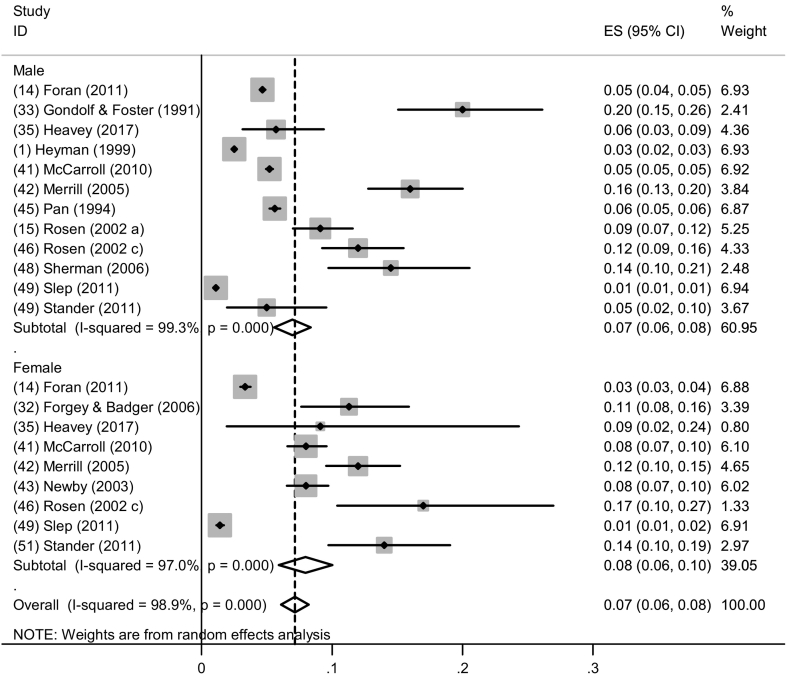
Forest plot of past year severe IPV perpetration by gender.

Twelve studies reported prevalence of past-year severe IPV perpetration with estimates ranging from 1.0%–20.0% (Foran et al., 2011; Forgey & Badger, 2006; Gondolf & Foster, 1991; Heavey et al., 2017; Heyman & Neidig, 1999; McCarroll et al., 2010; Merrill et al., 2005; Newby et al., 2003; Rosen, Knudson, et al., 2002; Sherman et al., 2006; Slep et al., 2011; Stander et al., 2011). All studies were included in the meta-analysis. The pooled prevalence was 7.0% (95% CI: 6.0%–8.0%, I^2^ = 98.1%, p < 0.001) (Fig. 3).

All twelve studies reported estimates of past-year severe IPV perpetration among men (Foran et al., 2011; Gondolf & Foster, 1991; Heavey et al., 2017; Heyman & Neidig, 1999; McCarroll et al., 2010; Merrill et al., 2005; Pan et al., 1994; Rosen, Knudson, et al., 2002; Rosen, Parmley, et al., 2002c; Sherman et al., 2006; Slep et al., 2011; Stander et al., 2011), and nine among women (Foran et al., 2011; Forgey & Badger, 2006; Heavey et al., 2017; McCarroll et al., 2010; Merrill et al., 2005; Newby et al., 2003; Rosen, Parmley, et al., 2002c; Slep et al., 2011; Stander et al., 2011). The pooled prevalence for men and women was 7.0% (95% CI: 6.0%–8.0%, I^2^ = 99.3%, p < 0.001) and 8.0% (95% CI: 6.0%–10.0%, I^2^ = 97.0%, p < 0.001) respectively (Fig. 3). While most of the studies reported prevalences between 3.0% and 17.0% among men and women, one study reported a higher prevalence of 20.0% among a sample of male Veterans in an alcohol detox facility (Gondolf & Foster, 1991). Three studies reported much lower estimates (1.1% among men (Slep et al., 2011), and 1.4% and 3.3% among women (Foran et al., 2011; Slep et al., 2011)). These studies were all conducted among AD Air Force personnel in general military settings.

Although there was no apparent difference between males and females when comparing the pooled prevalences, four studies reported higher prevalence of past-year self-reported severe IPV perpetration among women compared to men within the same study (8.0% vs. 5.2% (McCarroll et al., 2010); 14.0% vs. 5.0% (Stander et al., 2011); 9.1% vs. 5.7% (Heavey et al., 2017); 12% vs. 17% (Rosen, Parmley, et al., 2002c)). Two studies reported the opposite, higher prevalences of past-year severe IPV were seen among men compared to women within the same study (5.0% vs. 3.0% (Foran et al., 2011); 16.0% vs. 12.0% (Merrill et al., 2005)).

### 3.4 Sexual IPV perpetration

Five studies measured past-year sexual IPV perpetration (Forgey & Badger, 2006; Heavey et al., 2017; Kar & O'Leary, 2013; Teten et al., 2009; Tharp et al., 2016) and estimates ranged from 12.1%–40.2%. All studies used the CTS and/or CTS2 as a measure of sexual violence. Four studies reported estimates among men (Heavey et al., 2017; Klaw et al., 2016; Teten et al., 2009; Tharp et al., 2016). Three studies were conducted among Veteran populations. Two reported similar prevalence among samples of male US Iraq/Afghanistan Veterans (Klaw et al., 2016; Tharp et al., 2016), while the other was much higher among a clinical sample of US Veterans from a VA medical centre outpatient trauma recovery clinic (Teten et al., 2009). One study on female AD female Army personnel reported prevalences of 19.8% for minor sexual IPV perpetration (e.g. “insisting on sex when my partner did not want to”) and 0.4% for severe sexual IPV perpetration (e.g. “used force to make my partner have sex”) (Forgey & Badger, 2006; Straus, Hamby, Boney-McCoy, & Sugarman, 1996). One study directly compared men and women and found that among Army Reserve personnel, higher prevalence of men reported perpetrating sexual IPV compared women (14.9% and 12.1% respectively) (Heavey et al., 2017). Studies were too heterogeneous to conduct a meta-analysis.

### 3.5 Psychological/emotional IPV perpetration

Fifteen studies measured psychological/emotional IPV perpetration (a form of control used by IPV perpetrators to gain power over their partner by limiting their future options or freedom of choice). Eight studies measured past-year psychological/emotional IPV perpetration (Byrne & Riggs, 1996; Dutra et al., 2012; Forgey & Badger, 2006; LaMotte et al., 2017; Owens et al., 2013; Portnoy et al., 2018; Taft et al., 2009; Teten et al., 2009; Teten et al., 2010; Tharp et al., 2016), four studies measured IPV in the past 6-months (LaMotte et al., 2017; LaMotte, Taft, Reardon, & Miller, 2014; LaMotte, Taft, Weatherill, et al., 2014; Portnoy et al., 2018), one during the lifetime (Klaw et al., 2016) and two during the current relationship (Taft et al., 2007; Zamorski & Wiens-Kinkaid, 2013). Among males, prevalence estimates of past-year psychological IPV perpetration ranged from 66.4%–91.3% across seven studies (Byrne & Riggs, 1996; Klaw et al., 2016; Owens et al., 2013; Taft et al., 2009; Teten et al., 2009; Teten et al., 2010; Tharp et al., 2016). Among women, prevalence estimates of past-year psychological IPV perpetration ranged from 62.5%–89.5% across three studies (Dutra et al., 2012; Forgey & Badger, 2006; Owens et al., 2013).

A Canadian study comparing emotional/financial abuse perpetrated during the current relationship of male and female AD Regular personnel reported similar estimates among men and women (19.4% and 18.8% respectively) (Zamorski & Wiens-Kinkaid, 2013). A study that examined lifetime IPV among male US Iraq/Afghanistan Veterans did not provide an overall prevalence rate for psychological/emotional IPV perpetration but reported three separate estimates. 68% reported having insulted or sworn at their partner, 67.0% reported having shouted at their partner, 10.0% reported having destroyed something of their partner's (Klaw et al., 2016). A study that examined past-year IPV perpetration among a clinical sample of male Veterans found that 66.4% reported psychological aggression (Owens et al., 2013). Among another sample of male combat Veterans in a clinical setting, 91.3% reported psychological aggression (Taft et al., 2009). Most of the emotional abuse was described as put downs and name-calling. As the outcomes reported in the included studies were too heterogeneous, a meta-analysis was not conducted.

### 3.6 Serving status

Of the twenty-two studies that examined past-year physical IPV perpetration, nine were among AD personnel and thirteen among Veterans. Two studies did not stratify by gender (Buchholz et al., 2017; Kelley et al., 2015). Prevalence estimates among AD males (8 studies) ranged from 5.0%–32.0% (Foran et al., 2011; Heavey et al., 2017; Owens et al., 2013; Pan et al., 1994; Rosen, Knudson, et al., 2002; Rosen, Parmley, et al., 2002c; Smith Slep et al., 2010; Stander et al., 2011) and among AD women (6 studies) estimates ranged from 9%–38% (Foran et al., 2011; Forgey & Badger, 2006; Heavey et al., 2017; Rosen, Parmley, et al., 2002c; Smith Slep et al., 2010; Stander et al., 2011). Eleven studies of Veterans reported estimates among men ranging from 12%–57.6% (Byrne & Riggs, 1996; Gerlock et al., 2016; Hiley-Young et al., 1995; Hundt & Holohan, 2012; Kar & O'Leary, 2013; Klaw et al., 2016; Sherman et al., 2006; Taft et al., 2009; Teten et al., 2009; Teten et al., 2010; Tharp et al., 2016) and two reported estimates among women around 20% (Dutra et al., 2012; Hundt & Holohan, 2012).

**Fig. 4:**
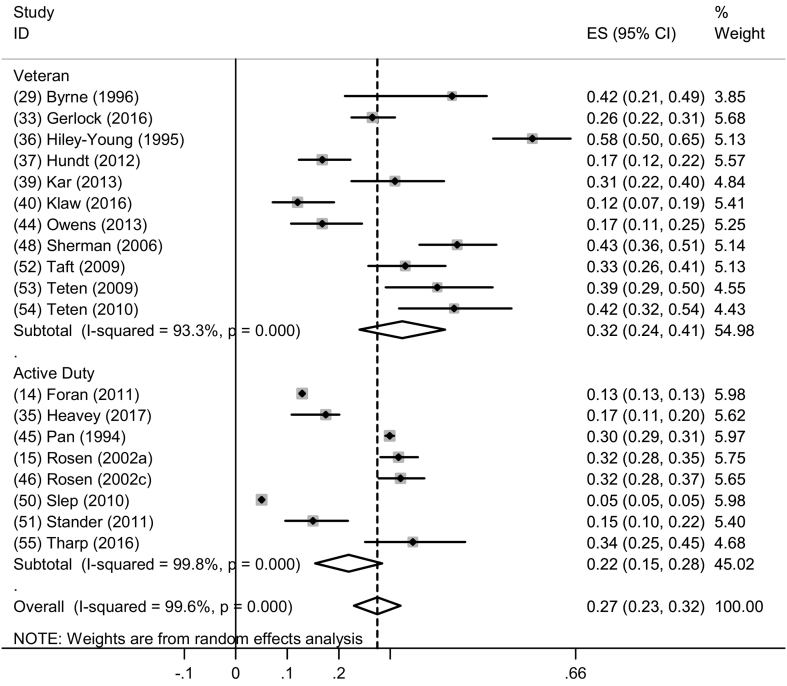
Forest plot of past year physical IPV perpetration by serving status among men.

A meta-analysis for past-year perpetrated physical IPV among men with a subgroup analysis by serving status was conducted as there were fourteen eligible studies. Seven studies reported estimates among AD personnel (Foran et al., 2011; Heavey et al., 2017; Pan et al., 1994; Rosen, Knudson, et al., 2002; Rosen, Parmley, et al., 2002c; Smith Slep et al., 2010; Stander et al., 2011) and eight among Veterans (Gerlock et al., 2016; Hiley-Young et al., 1995; Hundt & Holohan, 2012; Kar & O'Leary, 2013; Klaw et al., 2016; Owens et al., 2013; Sherman et al., 2006; Taft et al., 2009). The pooled prevalence of physical IPV perpetration among AD personnel was 22.0% (95% CI: 15%–28%; I^2^ = 99.8, *p* < 0.001) and among Veterans was 32.0% (95% CI: 24.0%–44.0%; I^2^ = 933.3%, p < 0.001) (Fig. 4). Only one eligible study examined past-year physical IPV perpetration among women Veterans (Hundt & Holohan, 2012) so a meta-analysis was not possible.

### 3.7 Branch of service and rank

Eight studies examined the prevalence of past-year physical IPV perpetration among men and women in different branches of service. Among males, prevalence estimates of past-year IPV perpetration varied by service among 6 studies. Three studies of Army personnel found prevalence estimates ranging from 29.9%–32.0% (Pan et al., 1994; Rosen, Knudson, et al., 2002; Rosen, Parmley, et al., 2002c). This was higher than among two studies of RAF with prevalences of 5.0% and 12.9% (Foran et al., 2011; Smith Slep et al., 2010) and one study of Navy personnel with a prevalence of 15.0% (Stander et al., 2011). Among females, two studies of Army personnel found prevalence estimates of 30.2% and 38.0% (Forgey & Badger, 2006; Rosen, Parmley, et al., 2002c), which were comparable to a study of Navy personnel which reported a prevalence of 32.0% (Stander et al., 2011), and higher than among two studies of RAF personnel which found prevalences of 9.0% and 15.1% (Foran et al., 2011; Smith Slep et al., 2010). Zamorski and colleagues (Zamorski & Wiens-Kinkaid, 2013) also reported a higher prevalence of physical/sexual IPV perpetration within the Army (10.7%) compared to the Air Force and Navy (8.3% and 8.8%, respectively) among both male and female Canadian Armed Forces personnel. However, the association between branch of service and physical or sexual IPV perpetration was not statistically significant when adjusted for confounding factors (Zamorski & Wiens-Kinkaid, 2013).

Two studies reported the prevalence of past-year physical IPV perpetration among males of different ranks (Foran et al., 2011; Rosen, Knudson, et al., 2002). Both reported higher prevalences among other ranks when compared to both NCOs and Officers. One study reported the prevalence of physical IPV by rank among women and found similar results of higher prevalence among lower ranks (Foran et al., 2011). Similar results of combined physical and/or sexual IPV perpetration were also found in a representative sample of Canadian Armed Forces personnel which found higher prevalences among the lower ranks. Another study compared past-year severe IPV perpetration among men and women of different ranks and also reported lower prevalences of past-year severe IPV perpetration among Officers and NCOs compared to other ranks (1.4%, 1.5% and 2.3%, respectively although not statistically different) (Foran et al., 2011).

### 3.8 Era of service

Data was collected during the pre-2001 period in seventeen studies and during the post-2001 period in twenty-one studies. Among men, eight studies provided prevalence estimates of past-year physical IPV perpetration pre-2001 ranging from 15.0%–57.6% (Byrne & Riggs, 1996; Hiley-Young et al., 1995; Pan et al., 1994; Rosen, Knudson, et al., 2002; Rosen, Parmley, et al., 2002c; Sherman et al., 2006; Stander et al., 2011; Teten et al., 2010) compared to 5.0%–47.0% among seven studies that used data collected during the post-2001 era (Foran et al., 2011; Heavey et al., 2017; Kar & O'Leary, 2013; Klaw et al., 2016; Smith Slep et al., 2010; Teten et al., 2010; Tharp et al., 2016). Among women, three studies provided prevalence estimates of past-year IPV perpetration during the pre-2001 era ranging from 22.0%–38.0% (Dutra et al., 2012; Rosen, Parmley, et al., 2002c; Stander et al., 2011) and four in the post-2001 era ranging from 9.0%–30.0% (Foran et al., 2011; Forgey & Badger, 2006; Heavey et al., 2017; Smith Slep et al., 2010).

**Fig. 5:**
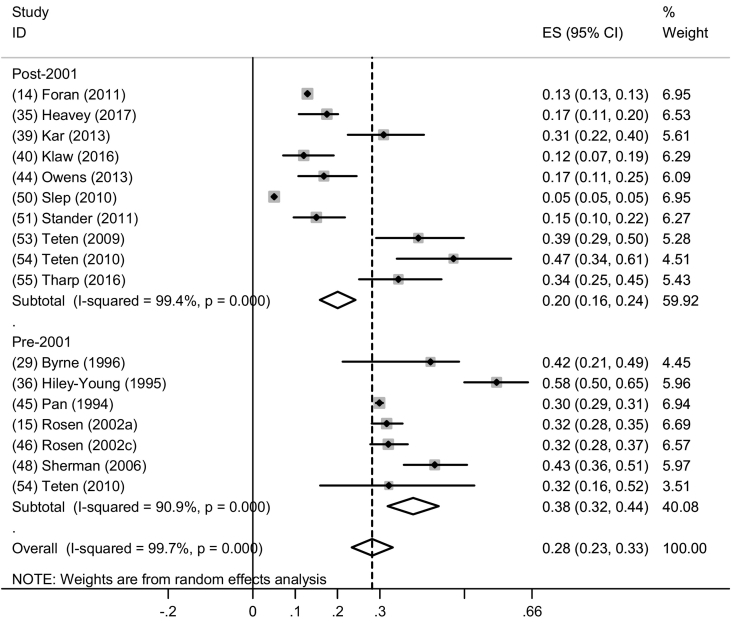
Forest plot of past year physical IPV perpetration by era of service among men.

Sixteen studies were eligible for inclusion in the meta-analysis examining era of service among men where the pooled prevalence of physical IPV perpetration pre-2001 was 38.0% (95% CI: 32.0%–44.0%; I^2^ = 90.9%, p < 0.001) across ten studies compared to 20.0% (95% CI: 16.0%–24.0%; I^2^ = 99.4%, p < 0.001) post-2001 across seven studies (Fig. 5). All but one study post-2001 were in general military settings – the study by Kar et al., with the highest prevalence, was in a clinical sample (Kar & O'Leary, 2013). Among studies in the pre-2001 era, four were conducted in general military settings and two (with higher prevalences) were in clinical settings (Hiley-Young et al., 1995; Sherman et al., 2006). Only one study pre-2001 was conducted among AD recruits (Stander et al., 2011) and this study reported the lowest prevalence of the subgroup. One study that directly compared past-year physical IPV perpetration between eras of service reported higher prevalence of past-year physical IPV perpetration among personnel in the post-2001 era compared to pre-2001 (47.4% vs. 32.1%, respectively) (Teten et al., 2010). There were not enough studies eligible for inclusion in a meta-analysis for past-year IPV perpetration among women.

### 3.9 Study setting

**Fig. 6:**
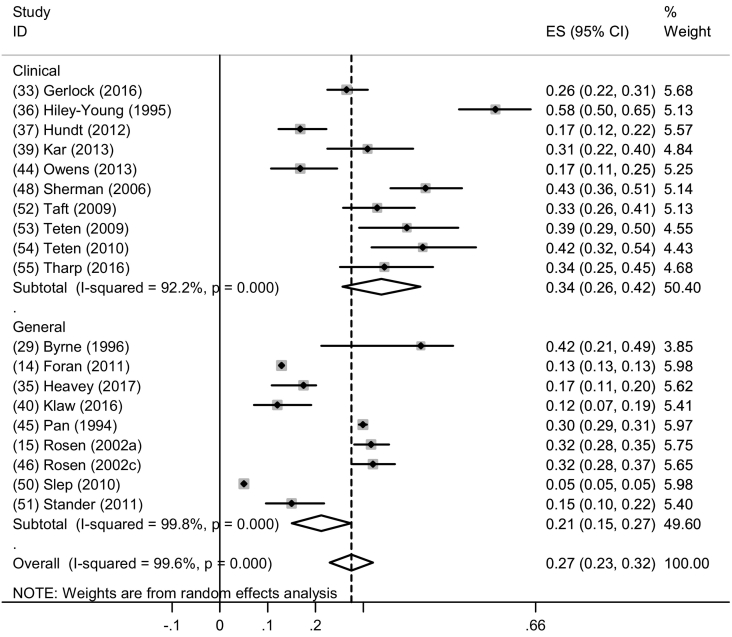
Forest plot of past year physical IPV perpetration by study setting among men.

Twenty-seven studies were conducted in general military settings and fifteen were conducted in clinical settings. The studies that measured past-year physical IPV perpetration provided the most number of studies by study setting (*n* = 14) (Fig. 6). The pooled prevalence of past-year physical IPV perpetration among men in general military settings (9 studies) was 21.0% (95% CI: 15.0%–27.0%, I^2^ = 99.8%, p < 0.001) compared to clinical populations of men (10 studies), where the pooled prevalence of physical IPV was 34.0% (95% CI: 26.0%–42.0%, I^2^ = 99.6%, p < 0.001). These results suggest higher prevalences of IPV reported in clinical settings compared to general settings.

## 4 Discussion

The aim of this review was to systematically review studies that estimate the prevalence of different types of IPV perpetration among serving and ex-serving military personnel. All included studies were conducted in North America. While most of the studies examined physical IPV perpetration, some also examined severe physical IPV, psychological/emotional IPV and sexual IPV perpetration. This review found conflicting results for gender bias in the prevalence of physical IPV perpetration, but no apparent gender differences were found for severe IPV perpetration. There appear to be gender differences in sexual IPV perpetration (higher prevalence among men), but none were found for psychological/emotional IPV perpetration. A consistently higher prevalence of all types of IPV perpetration was found among Veteran compared to AD samples, among the Army compared to other service branches and among the lower ranks compared to NCOs and Officers.

### 4.1 Prevalence of IPV among military populations

Several studies have suggested that prevalence of IPV perpetration is higher among military than civilian populations (Cronin, 1995; Griffin & Morgan, 1988; Heyman & Neidig, 1999). This review found that the prevalence of past-year physical IPV among men in general military population samples ranged from 5.0%–32.0% compared to studies in the US general population which found prevalences ranging from 4.0%–15.0% (Okuda et al., 2015; O'Leary, Tintle, & Bromet, 2014; Whitaker, 2014). Crude comparisons of prevalence among military populations and civilian populations are limited by lack of adjustment for sociodemographic factors that can impact on risk of IPV. Male predominance, relative youth and higher risk of heavy alcohol consumption are all factors that may increase the risk of IPV perpetration by military personnel (Fear et al., 2007; Wright, Foran, Wood, Eckford, & McGurk, 2012). It is likely that daily stressors of military life including frequent relocation or family separation may impact relationship satisfaction and may lead to higher rates of IPV (American Psychological Association Presidential Task Force on Military Deployment Services for Youth FaSM, 2007). A robust comparison of prevalence of IPV perpetration among military compared to civilian populations is needed with exploration of potential explanatory factors for any differences.

### 4.2 Impact of gender

Many studies examined IPV perpetration by male and/or female military personnel. While subgroup analyses showed higher prevalence of past-year physical IPV perpetration among men compared to women (26.0% vs. 20.0%, respectively), several studies directly compared males and females reported higher prevalences among the latter (Foran et al., 2011; Heavey et al., 2017; Rosen, Parmley, et al., 2002c; Smith Slep et al., 2010; Stander et al., 2011). These results are consistent with a study not included in the meta-analysis as it measured lifetime physical IPV perpetration (17.0% among men and 33.0% among women) (Waliski et al., 2013). Pooled prevalences of severe IPV perpetration were the similar among men and women in the meta-analysis (8.0% among men and 7.0% among women). However, among studies directly comparing males and females, the results were mixed, with some reporting higher prevalence of severe IPV perpetration among women compared to men (Heavey et al., 2017; McCarroll et al., 2010; Stander et al., 2011), and vice-versa (Foran et al., 2011; Merrill et al., 2005).

While most of the research in the general population focuses on IPV victimisation, there have been studies that examine IPV perpetration (Bohannon, Dosser, & Lindley, 1995; Houry et al., 2008; Tjaden & Thoennes, 2000). A meta-analysis of studies that reported acts of physical aggression during present or past relationships in the general population found little difference in the proportion of men and women who reported perpetrating physical aggression (Archer, 2002). Women were more likely to report having used one or more acts of physical aggression, but men were more likely to inflict injury on their partners (Archer, 2002). Walby et al. (2017) emphasise the discrepancy between perpetrator's actions and harms experienced by the victim. Often, the same action generates more harm/injury to the victim when perpetrated by a man compared to a woman (Walby et al., 2017). This review provides conflicting findings on the gender distribution of IPV among military populations, but unfortunately, only two of the included studies reported on the impact of perpetrated IPV (Foran et al., 2011; Slep et al., 2011) and therefore more research is needed to examine impact of perpetrated IPV among military personnel. In both studies, the prevalence of clinically significant IPV among men and women were similar suggesting that male and female military personnel are equally likely to perpetrate IPV resulting in injury.

Military culture has been viewed as an environment dominated by male machismo, in which commands and verbal aggression are part and parcel of everyday life (Anonymous, 2015; UNESCO Expert Group, 1997). Extrapolation of these attitudes and interpersonal styles into relationships and family life, or difficulty switching off when in the home environment, may be problematic and indeed the high prevalence of psychological/emotional IPV reported in the included studies seems to support this. Interestingly this appears to be a similar problem for both male and female military personnel. High levels of psychological/emotional abuse may also be influenced by high levels of stress and associated mental health problems (PTSD) which can be associated with angry (Jakupcak et al., 2007).

Few studies explored sexual IPV perpetration and of those that did, only one directly compared males and females and found a higher prevalence among males (Heavey et al., 2017). Most studies focused on male samples and found generally higher prevalences of sexual IPV perpetration (Klaw et al., 2016; Teten et al., 2009; Tharp et al., 2016). One study of sexual IPV perpetration among female military personnel reported lower prevalence of sexually assaultive behaviour than among males but the prevalence was still high at 20.0% (Forgey & Badger, 2006). It is important to note, however, that this study separated sexual violence into minor (19.8%) and severe (0.4%) sexual IPV perpetration. Given the more sensitive nature of this behaviour, under-reporting is a potential limitation of such studies, especially if surveys are not anonymous. High prevalences of sexual IPV perpetration among military personnel is in keeping with high rates of military sexual trauma (MST) that has been reported (Turchik & Wilson, 2010; Wilson, 2016). High rates of both sexual IPV perpetration and MST could be the result of factors that have been found to be associated with perpetrating sexual assaults in the general population – hyper-masculinity and alcohol use (Greathouse, Saunders, Matthews, Keller, & Miller, 2015).

As IPV is often referred to as “violence against women”, most research has focused on male perpetration and female victimisation. This review included three studies on purely female samples, and other studies that included women in their samples were either not disaggregated by gender or often had fewer than 20.0% women. The continued focus on women as victims and not as perpetrators of IPV in research will perpetuate the gap in the literature and preclude a better understanding of gender-based violence in the military.

#### 4.2.1 Impact of military characteristics

Studies among Veteran populations reported higher prevalence estimates compared to AD personnel. Results of subgroup analyses for past-year physical IPV were consistent with these findings. This is also consistent with research into violent behaviour among military personnel that has reported increased risk of violence among ex-serving compared to serving personnel (Kwan et al., 2018; MacManus et al., 2012; MacManus et al., 2013). However, six of the nine included studies on ex-serving personnel were conducted in clinical settings while all included studies on serving personnel were in general military settings. As mentioned above, studies in clinical settings reported higher prevalences compared to general settings. This may explain some or all the differences in prevalence of IPV perpetration between Veterans and AD personnel and contribute to the “healthy warrior effect” (Haley, 1998). Personnel who have issues requiring “treatment” in clinical settings are more likely to have been discharged leaving “healthy”, resilient personnel in AD serving status.

Another possible explanation is that Veterans have returned to civilian life and are home with their families and may have more opportunity to perpetrate IPV compared to AD personnel who are potentially not home or with their spouses as regularly. It is also possible that stressors associated with transition from military to civilian life may increase the risk for IPV perpetration (Elbogen et al., 2010). This may contribute to higher prevalence of past-year physical IPV perpetration among Veterans when compared to AD personnel.

Consistent with previous research into violent behaviour by military personnel (Kwan et al., 2017; MacManus et al., 2012), studies in this review found higher prevalence of IPV perpetration among Army personnel compared to Air Force and Navy personnel. As with research into violent behaviour, this could be a result of differences in sociodemographic factors. In general, Army personnel are younger males with lower levels of education (Department of Defense, 2014; Ministry of Defence, 2013), both of which are associated with increased risk of violent behaviour. Previous research has demonstrated characteristic differences between the branches of service (Kwan et al., 2017; Kwan et al., 2018; MacManus et al., 2012). Future studies should sample the entire military and compare branches of service within-study.

Studies that compared IPV prevalence by rank demonstrated higher prevalence among lower ranks (enlisted personnel) when compared to higher ranks (Officers). This is not surprising as research has consistently demonstrated that military personnel of lower ranks in both US and UK populations have higher risk of violent behaviour and offending often due to their sociodemographic characteristics (i.e. younger, single males with lower socioeconomic status) (Elbogen et al., 2010; Gallaway, Fink, Millikan, & Bell, 2012; MacManus et al., 2012).

### 4.3 Era of service

Studies which utilised data collected post-2001 generally reported lower prevalence of IPV perpetration than studies using data collected pre-2001. There are several possible interpretations of this finding. This may reflect a societal shift in awareness of IPV and growing campaigns to prevent IPV in the general population and Armed Forces (Department of Health and Human Services, 2016; Department of Justice Canada, 2002; HM Government, 2011). It may also reflect improved mental health support services for military personnel in the US since the end of the Vietnam era in response to growing awareness of the mental health consequences of conflict and the impact on families (Pols & Oak, 2007). These services may be helping military personnel cope with their mental health problems and, in turn, reduce the risk of IPV perpetration. Whilst this is an encouraging finding, further research is required to better understand factors underlying the apparent reduction in IPV perpetration over time.

### 4.4 Impact of study setting

Studies conducted in general military settings had lower prevalences of IPV perpetration compared to studies conducted in clinical settings. The samples used in the studies conducted in clinical settings were mainly patients with PTSD which has been shown in previous research to be associated with increased risk of violence among military personnel (Elbogen et al., 2014; Kwan et al., 2017; Kwan et al., 2018; MacManus et al., 2012).

### 4.5 Limitations of research

This review highlights that good quality research into IPV in the military is sparse. There were several well-designed studies with large, representative samples of military personnel, but many of the included studies used small selected clinical samples. An important limitation of the review is the poor comparability of included studies. Comparing the prevalence of IPV perpetration across studies is problematic given the different study methodologies. Different assessment tools have been used in different time periods. Many of the studies in this review used the CTS to measure violence, which has been criticised for not considering the context of abuse (i.e. whether the acts of violence were in attack or self-defence). This could lead to misclassification bias across genders. Some studies examined past-year IPV while others recorded lifetime IPV, within the current relationship or during any relationship.

While most of the studies used the CTS/ CTS2 (Straus, 2007) to measure IPV perpetration, some studies used other adapted measurement tools or their own measurement tools. The use of different measurement tools could contribute to the range of prevalences of IPV perpetration. One outlier was Hiley-Young (Hiley-Young et al., 1995) that reported a much higher prevalence of physical IPV perpetration which may have been the result of the use of their own measure of IPV by asking participants if they had reported violence towards their wife. It did not ask about specific items included on the CTS/CTS2. As no specific items were asked, anything that could be considered “violence” could have been included as reported IPV perpetration.

Of the included studies in this review, only two considered impact of perpetrated physical IPV (Foran et al., 2011; Slep et al., 2011). Impact of IPV is not only important to consider the different levels of physical injury sustained by the victim depending on the perpetrator's gender, but is also important to consider psychological/emotional impact/injury. While a female perpetrator may not be able to inflict as much physical injury on a male victim, the level of psychological/emotional injury may be high. Similarly, less serious, but consistent forms of physical violence by either gender (e.g. pushing/shoving) may not result in any physical injury, but may have a strong impact on the mental health of the victim. To effectively measure IPV, including impact, it is necessary to consider the event, perpetrator and victim (Walby et al., 2017). Therefore, it is important not only to consider the type of IPV being perpetrated, but also, by whom, and the impact the act may have on the victim.

As the studies included in this review were all conducted in North America (US and Canada), the results are not currently generalisable to UK and other international military populations. This suggests that more research on IPV perpetration among UK military populations is needed to begin to build an evidence base.

### 4.6 Implications

Despite the limitations of the studies included in this review, findings confirm previous research that IPV is prevalent among military populations and among both sexes. Although less researched than physical IPV, psychological/emotional IPV was universally found to be more prevalent than physical IPV. Concerns remain about attitudes to and awareness of IPV among military personnel and how much understanding there is of the diverse nature of IPV which includes controlling and emotionally and psychologically abusive behaviours as well as physical abuse (Marshall et al., 2005). This review provides evidence of the prevalence of these abuses and the need for Armed Forces to strengthen efforts and improve training and awareness opportunities. It highlights particularly “at risk” groups such as those in the Army, in lower ranks and who have left service. A better understanding is needed of the impact on the risk of IPV of military life and experiences unique to the military, such training in and exposure to combat, frequent transitions and trauma-related mental health problems.

## 5 Conclusions

Intimate partner violence has been shown to be a significant issue among military personnel. While gender differences exist, differences found within military populations are not as large as may be expected. Further examination is needed of gender distribution of IPV among military populations and future research should not only consider individual acts of IPV, but also the impact each act has on the victim. These findings will inform IPV prevention and management programs.

## Declarations of interest

None.

## Figures and Tables

**Table:** 

Part 1					
Screening questions			Score		
	Question	Comments	0	1	2
1	Did the study ask a clearly focused question? *– Is the hypothesis/aim/objective of the study clearly described?* *-Is the study question focused in terms of the outcomes considered?*				
2	Is the study design appropriate for the research question?				
3	Was a validated tool used to assess mental disorder? *-Diagnostic interview using validated instrument,* e.g.*, SCID =* *2* *-Screening instrument for mental disorder* e.g. *PHQ, GAD-7, CES-D*				
P					
Continue only if score on each of questions 1 and 2 is one or more					
**Detailed questions**					
Measurement of risk of selection bias					
4a	Is the sampling method appropriate for the research question? *Consider:* *-The sampling method used (*i.e. *random selection of subjects)* *- If applicable, is there appropriate selection of controls?*				
4b	Are subjects appropriately defined? *Consider:* *- Inclusion/ exclusion criteria specified* *- Inclusion/exclusion criteria appropriate*				
4c	Is the sample size appropriate? *Consider:* *- Is the sample size justified?* *- Were a sufficient number of cases selected?* *- If applicable, were a sufficient number of controls selected?*				
4d	Is the study sample representative of the population of interest? *-Do the authors assess the representativeness of the study sample?*				
4e	Does the level of non-participation risk introducing bias? *Consider:* *-Are key demographic characteristics of non-participants reported and compared against participants?* *-Does the study report on the impact of non-participation?* *-If applicable, rates of attrition reported*				
5	Is the study setting appropriate to the aims of the research? (e.g. setting, location, relevant dates)				
6	Is the method of data collection appropriate for the aims of the research?				

Measurement of risk of reporting bias					
7	Are suitable/standard criteria used for measurement of domestic violence? *Consider:* *-Criteria of domestic violence was clearly defined* *-Potential for bias of measurement* *-If measures piloted* *- Standardised/pre-validated measures (score 2 points)* *- Researchers developed their own measure (score 1 point)* *- No details of measurement were provided (score 0 point)*				
8	Are known confounders accounted for by study design? *- Was consideration of confounding factors accounted for in study design?*				
9	Are known confounders accounted for in the analyses?				
10	Are the statistical tests used to assess the main outcomes appropriate? *-Was there adequate adjustment for confounding in the analyses?* *- Do the analyses adjust for different lengths of follow-up (if applicable)?*				
11a	Are the estimates reported with confidence intervals and in detail by sub-group (if appropriate)? *- Were the findings reported clearly?*				
11b	Are statistically non-significant results presented?				
11c	Are data for relevant variables complete?				
12	Was the conduct of the fieldwork appropriate to the study setting? *-Was the allocation of the interviewer/interpreter sensitive to the background of the participant?* *-Were fieldworkers trained and supported to work with people who have perpetrated domestic violence?*				
13	Were ethical considerations appropriately considered? -*Did researchers obtain informed consent from all participants?* *- Did researchers take adequate precautions to safeguard participants' anonymity and confidentiality?* *-Did fieldworkers offer information about domestic violence support and referral options to all participants?* -*Were fieldworkers appropriately trained to deal with participant distress?*				
14	Do the findings support the conclusions?				
15	Are the strengths and weaknesses of the research discussed?				

Calculate total score (out of a possible total of 42):______

## Appendix A Search terms - example search in EMBASE

Service personnel and veterans 1 or 2 or 3 or 4 or 5 or 6 or 7 or 8 or 9 or 10 or 11 or 12 or 13 or 14 = 9,208.

1. Veterans/
2. (air adj1 forc$).ti,ab.
3. (royal adj1 air adj1 forc$).ti,ab.
4. ((navy or naval) adj1 personnel).ti,ab.
5. exp enlisted military personnel/
6. exp military personnel/
7. exp military personnel/
8. (military adj1 person$).ti,ab.
9. (army adj1 person$).ti,ab.
10. (armed adj1 force$).ti,ab.
11. (coast adj1 guard).ti,ab.
12. (national adj1 guard).ti,ab.
13. reservist$.mp. [mp=title, abstract, original title, name of substance word, subject heading word, keyword heading word, protocol supplementary concept word, rare disease supplementary concept word, unique identifier]
14. (reserve adj1 force$).ti,ab.

Intimate partner 1 or 2 or 3 or 4 or 5 or 6 or 7or 8 or 9 = 40,924

1. exp husbands/ or exp spouses/
2. exp wives/ or exp spouses/
3. romance/ or exp couples/ or exp significant others/
4. (intimate adj1 (partner$ or couple)).mp. [mp=title, abstract, original title, name of substance word, subject heading word, keyword heading word, protocol supplementary concept word, rare disease supplementary concept word, unique identifier]
5. girlfriend.mp. [mp=title, abstract, original title, name of substance word, subject heading word, keyword heading word, protocol supplementary concept word, rare disease supplementary concept word, unique identifier]
6. boyfriend.mp. [mp=title, abstract, original title, name of substance word, subject heading word, keyword heading word, protocol supplementary concept word, rare disease supplementary concept word, unique identifier]
7. (romantic adj1 (partner$ or couple$)).mp. [mp=title, abstract, original title, name of substance word, subject heading word, keyword heading word, protocol supplementary concept word, rare disease supplementary concept word, unique identifier]
8. (other adj1 half).mp. [mp=title, abstract, original title, name of substance word, subject heading word, keyword heading word, protocol supplementary concept word, rare disease supplementary concept word, unique identifier]
9. exp dyads/ or exp couples/

Intimate partner violence 1 or 2 or 3 or 4 = 4,803

1. domestic violence/ or exp battered females/ or exp emotional abuse/ or exp intimate partner violence/ or exp marital conflict/ or exp partner abuse/ or exp physical abuse/ or exp sexual abuse/
2. ((abuse or batter$ or violen$ or beat$) adj2 (domestic or partner or spouse or woman or women or men or man or female$ of male or wife or wives or husband$ or boyfriend$ or girlfriend$)).ti,ab.
3. (((domestic adj1 violence) or intimate) adj1 partner adj1 violence).mp. [mp=title, abstract, original title, name of substance word, subject heading word, keyword heading word, protocol supplementary concept word, rare disease supplementary concept word, unique identifier]
4. (domestic adj1 homicid$).mp. [mp=title, abstract, original title, name of substance word, subject heading word, keyword heading word, protocol supplementary concept word, rare disease supplementary concept word, unique identifier]

Prevalence 1 or 2 or 3 or 4 or 5 or 6 = 4,622,284.

1. exp Epidemiology/ or exp Risk Factors/
2. incidence.mp. [mp=title, abstract, original title, name of substance word, subject heading word, keyword heading word, protocol supplementary concept word, rare disease supplementary concept word, unique identifier]
3. rates.mp. [mp=title, abstract, original title, name of substance word, subject heading word, keyword heading word, protocol supplementary concept word, rare disease supplementary concept word, unique identifier]
4. exp population/ or exp statistics/
5. population statistics.mp. [mp=title, abstract, original title, name of substance word, subject heading word, keyword heading word, protocol supplementary concept word, rare disease supplementary concept word, unique identifier]
6. exp statistical data/

[Service personnel and veterans terms] AND [Intimate Partner terms] AND [Intimate Partner Violence terms] AND [Prevalence terms] = 42.

## Appendix B Quality appraisal form

Please complete part 1 for all study designs and complete the relevant sections for part 2, specific to study design.

Score the answer to each question by ticking 0, 1 or 2:

0 – study does not meet criteria/answer question.

1 – Study partially meets criteria/gives a partially satisfactory answer to the question.

2 – Study fully meets criteria/gives a fully satisfactory answer to the question.
